# Investigation of the Potential of Heterophil/Lymphocyte Ratio as a Biomarker to Predict Colonization Resistance and Inflammatory Response to *Salmonella enteritidis* Infection in Chicken

**DOI:** 10.3390/pathogens11010072

**Published:** 2022-01-07

**Authors:** Mamadou Thiam, Astrid Lissette Barreto Sánchez, Jin Zhang, Jie Wen, Guiping Zhao, Qiao Wang

**Affiliations:** State Key Laboratory of Animal Nutrition, Institute of Animal Sciences, Chinese Academy of Agricultural Sciences, Beijing 100193, China; mamadou.sleam@gmail.com (M.T.); asliss_07@hotmail.com (A.L.B.S.); zhangjin0913@126.com (J.Z.); wenjie@caas.cn (J.W.); zhaoguiping@caas.cn (G.Z.)

**Keywords:** chicken, H/L, performance, colonization resistance, ROD21 pathogenicity island, inflammatory response, *Salmonella*

## Abstract

*Salmonella* causes significant economic loss to the poultry industry and represents a real threat to human health. The region of difference 21 (ROD21) pathogenicity island removal is a genetic mechanism by which *Salmonella* *enteritidis* (SE) invades the intestinal epithelium and induces systemic infection in mice. The heterophil/lymphocyte (H/L) ratio reflects the chicken’s robustness and immune system status. The H/L ratio is considered a disease resistance trait, and it could be used as a marker for selecting *Salmonella* resistance in live chickens. However, the association of the H/L ratio with *Salmonella* resistance and the inflammatory response remains to be elucidated. Moreover, the kinetics of ROD21 excision in the intestine and immune organs of chickens is unknown. Therefore, this study aimed to investigate the bacterial load, the ROD21 excision, the IL-1β, IL-8, and INF-γ blood serum concentration kinetics, and the association with the H/L ratio in chicken at 1, 3, 7, and 21 days post-SE infection. The results showed a significant correlation between the H/L ratio and the bacterial load in the ileum and caecum at 7 dpi. The ROD21 pathogenicity island absolute and relative excision in the caecum were positively correlated at 1 dpi but negatively correlated at 7 dpi with the H/L ratio. However, in the liver, we found the opposite tendency. The association of the H/L ratio with IL-1β, IL-8, and INF-γ blood serum concentrations showed that a low H/L ratio is correlated with increased IL-1β and INF-γ at 21 dpi. This study confirmed that the H/L ratio is associated with robustness and *Salmonella*-resistance in chicken. The methodology used in this study can separate individuals into susceptible and resistant and can help in the selection and breeding of *Salmonella*-resistant chickens.

## 1. Introduction

*Salmonella* and *Campylobacter* are responsible for most food safety concerns related to chicken production [1]. Salmonellosis in poultry and humans is responsible for significant morbidity and death during disease outbreaks [2]. *Salmonella* can be transmitted vertically through contaminated breeding flocks, horizontally through infected subpopulations of birds within a flock, or from environmental factors [3]. Such a disease negatively impacts the poultry industry and poses a menace to public health. Current measures include vaccination and antibiotic drugs. However, antibiotic therapy can lead to the emergence of resistance, acute diarrhea, and possible chronic inflammation, which constitute a significant concern for the poultry industry [4]. Accordingly, the pursuit of alternatives to antibiotic treatment [5] has become the object of considerable research interest [6]. Unfortunately, despite the implementation of a range of feed additives, including probiotics, prebiotics, phytogenics, and chemicals, such as organic acids [6,7,8,9,10], salmonellosis continues to be a threat for the poultry production [11]. Thus, one of the primary goals in chicken breeding is to improve the resistance of chickens against *Salmonella* infection by selecting and creating disease-resistant chicken lines.

Region Of difference 21 (ROD21) is an excisable pathogenicity island found on the chromosomes of *Salmonella enteritidis*, *Salmonella gallinarum*, *Salmonella dublin*, and *Salmonella typhi* [12]. The presence of ROD21 has been shown to enhance liver and spleen colonization in mice [13]. Furthermore, it was recently revealed that ROD21 excision is a genetic mechanism required for *Salmonella enteritidis* to invade the intestinal epithelium successfully, a critical step for systemic infection in mice [14]. However, despite the rapid development of immunological technologies and their applications in poultry, no studies assessed the kinetic of ROD21 excision in chickens or wild avian species.

Heterophils and lymphocytes are the two most abundant white blood cell types in birds, playing an essential role in innate and adaptative immunity, respectively [15]. The H/L ratio may reflect a readiness to cope with infection through injury (via heterophils) rather than with a communicable disease (via lymphocytes) [15]. Likewise, the H/L ratio in the blood reflects the immune system status [16,17]. Moreover, it has been used as a selection criterion for the Newcastle disease vaccine’s response, general resistance to heat stress [18], and *Salmonella typhimurium* resistance [19]. Several reports indicated that this trait is highly heritable in poultry [19,20] and wild avian species [21].

Interferon-gamma (IFN-γ) and interleukins (ILs) are critical components of the immune system. Moreover, these molecules are a vital link between the innate and adaptive immune systems [22]. For example, *Salmonella* infection generally decreases the level of IL-1β, IL-8, and IFN-γ in the plasma [23], but increases their *mRNA* expression levels in the small intestine [24]. However, the link between the H/L ratio and these blood serum immune indicators remains unclear.

The H/L ratio has been extensively studied to select poultry resistant to environmental stressors. However, to our knowledge, no studies have linked this trait with the resistance and inflammatory response to *Salmonella* infection in chicken. Therefore, the objectives of the present study were to (1) determine the effects of *Salmonella enteritidis* infection on the live weight, colonization resistance in the blood and different tissues, and inflammatory response in the blood serum, and (2) to assess the relationship between the H/L ratio at 7 days old with the colonization resistance and inflammatory response to *Salmonella* infection during the infective cycle (1, 3, 7, and 21 dpi). This study provides valuable resources for the fast diagnosis and development of more specific technics to control salmonellosis. Furthermore, it may facilitate the breeding of disease-resistant chickens.

## 2. Results

### 2.1. Effects of Salmonella Enteritidis Infection on the Survival Rate and Live Weight Post-Infection

**Figure 1:**
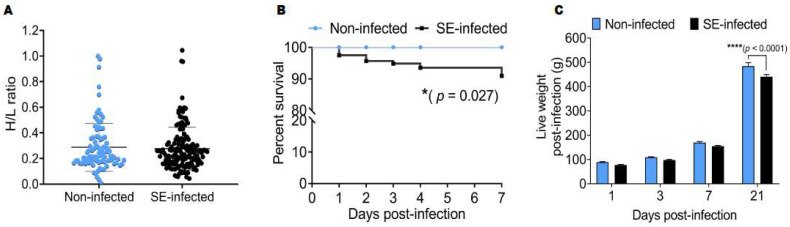
H/L ratio comparison before challenge and effects of *Salmonella* challenge on the survival rate and live weight post-infection. (**A**) H/L ratio of non-infected (n = 92) and SE-infected (n = 159) chickens group at 7 days old (before challenge). (**B**) Survival curve Kaplan–Meier comparing non-infected and SE-infected groups during the first week post-infection. (**C**) Live weight post-infection comparison between non-infected and SE-infected chickens (n = 16). Two-way ANOVA analyzed weight comparisons between non-infected and SE-infected during the infective cycle with Sidak’s multiple comparisons test. * *p* < 0.05 and **** *p* < 0.0001.

Before the challenge, the H/L ratio was measured between the two groups of chickens, and the results showed that the H/L ratios of non-infected (0.29 ± 0.19) and SE-infected (0.28 ± 0.17) chickens at 7 days old (before infection) were not significantly different (Figure 1A). The survival rate between the two groups recorded during the first week after the *Salmonella* challenge was significantly different (*p* < 0.05). Remarkably, 100% of non-infected (NI) chickens survived, whereas only 90.94% of SE-infected (SE) chickens survived after *Salmonella* infection (Figure 1B). Since the H/L ratio has emerged as an important indicator of robustness, we also investigated the effects of the *Salmonella enteritidis* challenge on the live weight (at 1, 3, 7, and 21 dpi). Remarkably, only at 21 dpi, the live weight of NI (484.30 ± 56.19 g) chickens was significantly (*p* < 0.0001) increased, compared to SE (440.94 ± 35.14 g) chickens (Figure 1C).

### 2.2. Differences in Salmonella Enteritidis Load in the Blood, Ileum, Caecum, and Liver at 1, 3, 7, and 21 Days after Infection, and Association with H/L Ratio

**Figure 2:**
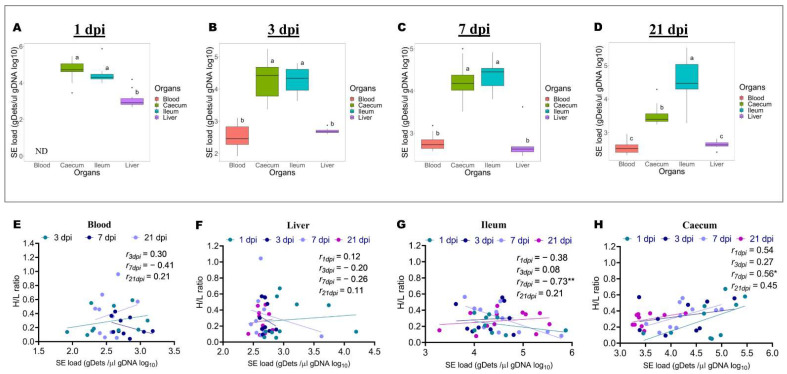
The bacterial load in the blood, liver, ileum, and caecum differs significantly during the infective cycle, and the bacterial loads in the ileum and caecum at 7 dpi are significantly and inversely correlated to the H/L ratio. (**A**–**D**) represent the bacterial among the blood, liver, ileum, and caecum at 1, 3, 7, and 21 dpi, respectively, except the bacterial load in the blood at 1 dpi, which was not determined (n = 10). (**E**–**H**) represent the correlation between the H/L ratio and the bacterial load in the blood, liver, ileum, and caecum, respectively. Comparisons of bacterial loads between different organs were analyzed by one-way ANOVA with Tukey’s post-test, and the correlations between the H/L ratio and the bacterial load were analyzed by Pearson correlation with Student’s *t*-test (one tail). ^a,b^ Means at each time point post-infection with no common superscript indicate significant difference (*p* < 0.05). * *p* < 0.05 and ** *p* < 0.01.

To support the notion that the H/L ratio at 7 days old can be used as an immune indicator for selecting chickens resistant to *Salmonella* infection, the bacterial load from the blood, liver, ileum, and caecum was determined by Q-PCR. Further, the relationship between the H/L ratio and the bacterial load in different organs was assessed. As shown in Figure 2A–D, the SE load was detected in the blood, liver, ileum, and caecum at 1, 3, 7, and 21 dpi, except for the blood, which was not determined at 1 dpi. It was noteworthy that the bacterial load calculated in these different organs during the infective cycle varied between 2.71 to 4.70 gDets/µL gDNA. Moreover, the bacterial load was increased in the intestine compared to that in the blood and liver. At 1 dpi, the results were 4.70 gDets/µL gDNA, 4.46 gDets/µL gDNA, and 3.10 gDets/µL gDNA for the caecum, ileum, and liver, respectively (Figure 2A). Tukey’s test showed that the bacterial load in the caecum and ileum were comparable but significantly (*p* < 0.0001) increased in comparison to the bacterial load in the liver at 1 dpi (Figure 2A). At 3 dpi, the results were 4.29 gDets/µL gDNA, 426 gDets/µL gDNA, 2.70 gDets/µL gDNA, and 2.53 gDets/µL gDNA for the ileum, caecum, liver, and blood, respectively (Figure 2B). Similarly, to 1 dpi, at 3 dpi, the bacterial load in the caecum and ileum were comparable, but significantly (*p* < 0.0001) increased in comparison to the bacterial load in the liver and the blood, which were similar (Figure 2B). At 7 dpi, the results were 4.46 gDets/µL gDNA, 4.23 gDets/µL gDNA, 2.79 gDets/µL gDNA, and 2.71 gDets/µL gDNA for the ileum, caecum, blood, and liver, respectively (Figure 2C). The Tukey’s test showed the same results obtained at 3 dpi, despite a numerical difference between the ileum and caecum bacterial load (Figure 2C). At 21 dpi, the results were 4.57 gDets/µL gDNA, 3.52 gDets/µL gDNA, 2.64 gDets/µL gDNA, and 2.55 gDets/µL gDNA for the ileum, caecum, liver, and blood, respectively (Figure 2D). Tukey’s test revealed that, at this time point, the bacterial load was significantly (*p* < 0.0001) increased in the ileum compared to that of the caecum, which was significantly (*p* < 0.0001) increased compared to that of the liver and the blood, which were comparable (Figure 2D). The correlation analysis between the H/L ratio and the bacterial load in each organ is shown in Figure 2E–H. The results showed no significant correlation between the H/L ratio and the bacterial load in the blood and liver during the infective cycle (Figure 2E,F). However, at 7 dpi, the H/L ratio was significantly and negatively correlated (r = −0.73 and *p* < 0.01) to the bacterial load in the ileum (Figure 2G). In contrast, it was significantly and positively correlated (r = 0.56 and *p* < 0.05) with the bacterial load in the caecum (Figure 2H).

### 2.3. ROD21 Absolute and Relative Excision Kinetic, and Association with H/L Ratio

**Figure 3:**
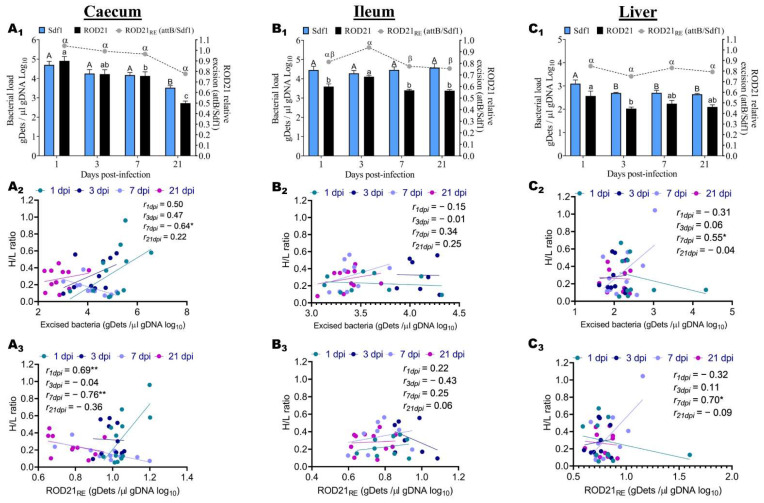
Determination of ROD21 excision during the infective cycle and association with the H/L ratio. After infection of the chickens with *Salmonella enteritidis*, the quantification of total *Salmonella* gDets and absolute ROD21 excision in the caecum, ileum, and liver was performed by quantifying the Sdf1 (total bacteria, blue bars) or attB (excised bacteria, black bars) sequences by qPCR. The gDNA from caecum (**A_1_**), ileum (**B_1_**), and liver (**C_1_**) were purified and the number of copies for each sequence per μL of gDNA was determined. The grey circles and continued line over each instance of post-infection are the relative ROD21 excision values (ROD21_RE_ = attB gDets/Sdf1 gDets). (**A_2_**,**A_3_**) represent the association of the H/L ratio with ROD21 absolute and relative excision in the caecum, respectively. (**B_2_**,**B_3_**) represent the association of the H/L ratio with ROD21 absolute and relative excision in the ileum, respectively. (**C_2_**,**C_3_**) represent the association of the H/L ratio with ROD21 absolute and relative excision in the liver, respectively. The assay included 10 chickens for each time, except for the ileum, which included 7 to 10 chickens. ^A,B/a,b/α,β^ Means of different time points post-infection with no common superscript indicate significant difference (*p* < 0.05). One-way ANOVA with Tukey’s post-test (α = 0.05) was used to compare different time points post-infection. The correlations between the H/L ratio and the bacterial load were analyzed by Pearson correlation with Student’s t-test (one tail). * *p* < 0.05 and ** *p* < 0.01.

In the same perspective, in order to establish the relationship between the H/L ratio and the colonization resistance, we assessed the frequency of excision of ROD21 in the liver and different portions of the gastrointestinal tract (ileum and caecum) during the infective cycle. First, the Sdf1 sequence was used to detect the total number of bacteria and attB was used to detect the bacteria that underwent excision. Then, we assessed the excision frequency by calculating the ROD21 relative excision (ROD21_RE_ = attB gDets/Sdf1 gDets). We observed that the ROD21 absolute and relative excision calculated in these tissues varied from 2.02 to 4.91 gDets/µL gDNA and from 0.75 to 1.04, respectively (Figure 3A_1_,B_1_,C_1_). Moreover, we observed that the number of *Salmonella enteritidis* that underwent excision was increased in the caecum (Figure 3A_1_) and ileum (Figure 3B_1_) compared to that of the liver (Figure 3C_1_). Notably, the relative ROD21 excision observed at 3 dpi in the ileum (Figure 3B_1_) was significantly increased and almost equivalent to that observed at 1 dpi in the caecum (Figure 3A_1_).

The total number of *Salmonella* detected in the caecum at 1, 3, 7, and 21 dpi was 4.70 gDets/µL gDNA, 4.26 gDets/µL gDNA, 4.17 gDets/µL gDNA, and 3.52 gDets/µL gDNA, respectively, and the Tukey’s test showed that the bacterial loads at 1, 3, and 7 dpi were significantly (*p* < 0.0001) increased, compared to that of 21 dpi (Figure 3A_1_). In the ileum, the results at 1, 3, 7, and 21 dpi were 4.46 gDets/µL gDNA, 4.29 gDets/µL gDNA, 4.46 gDets/µL gDNA, and 4.58 gDets/µL gDNA, respectively, and the multiple comparisons showed no significant difference among the different time points (Figure 3B_1_). In the liver, the bacterial loads at 1, 3, 7, and 21 dpi were 3.10 gDets/µL gDNA, 2.71 gDets/µL gDNA, 2.70 gDets/µL gDNA, and 2.64 gDets/µL gDNA, respectively, and the Tukey’s test showed that the bacterial load at 1 dpi was significantly (*p* < 0.0001) increased in comparison to that of 3, 7, and 21 dpi, which were comparable among them (Figure 3C_1_). After determining the total number of *Salmonella*, we detected the number of excised bacteria in the different tissues pre-cited during the infective cycle.

The number of *Salmonella enteritidis* that underwent ROD21 excision (ROD21) at 1, 3, 7, and 21 dpi was 4.91, 4.23, 4.13, and 2.72 respectively for the caecum, and the Tukey’s multiple comparisons revealed that the ROD21 at 1 dpi was significantly increased, compared to that of 3, 7, and 21 dpi (Figure 3A_1_). However, ROD21 at 3 dpi was comparable to that of 1 dpi and 7 dpi and significantly increased compared to 21 dpi (Figure 3A_1_). From the ileum, the number of excised bacteria at 1, 3, 7, and 21 dpi was 3.60, 4.11, 3.40, and 3.38 respectively, and the multiple comparisons showed that ROD21 at 3 dpi was significantly increased compared to that of 1, 7, and 21 dpi, which were comparable among them (Figure 3B_1_). In the liver, the results of excised bacteria at 1, 3, 7, and 21 dpi were 2.57, 2.02, 2.24, and 2.10, respectively, and the multiple comparisons showed that the result at 1 dpi was significantly increased compared to that of 3, 7, and 21 dpi, which were comparable among them (Figure 3C_1_). Nevertheless, the results also showed that the number of excised bacteria at 7 and 21 dpi was equivalent (Figure 3C_1_). To establish the relationship between the H/L ratio and the colonization resistance, we calculated the relative ROD21 excision (ROD21_RE_) during the infective cycle. The ROD21_RE_ of *Salmonella* detected in the caecum at 1, 3, 7, and 21 dpi was 1.04, 0.99, 0.96, and 0.77, respectively, and the Tukey’s test showed no significant difference among the four time points post-infection (Figure 3A1). From the ileum, the ROD21_RE_ results at 1, 3, 7, and 21 dpi were 0.81, 0.94, 0.77, and 0.76, respectively, and the multiple comparisons showed that the ROD21_RE_ at 1 and 3 dpi was significantly increased compared to that of 7 and 21 dpi, which was comparable between them, and to that of 1 dpi (Figure 3B_1_). The results of ROD21_RE_ at 1, 3, 7, and 21 dpi were 0.85, 0.75, 0.83, and 0.79, respectively, for the liver, and the Tukey’s test showed no significant differences among the different time points (Figure 3C_1_). A correlation analysis of the H/L ratio with the total number of excised bacteria (ROD21) and the relative ROD21 excision (ROD21_RE_) from different tissues during the infective cycle was assessed, and the results are presented in Figure 3A_2_,A_3_,B_2_,B_3_,C_2_,C_3_. As expected, the H/L ratio was highly correlated to the ROD21 and ROD21_RE_. Moreover, it was remarkable that the association of the H/L ratio with the ROD21 and ROD21_RE_ in the caecum (Figure 3A_2_,A_3_) showed opposite trends to that of the liver (Figure 3C_2_,C_3_) at 1 and 7 dpi. The association of the H/L ratio with the ROD21 and ROD21_RE_ in caecum at 1, 3, 7, and 21 dpi showed that the H/L ratio was significantly and negatively correlated to ROD21 at 7 dpi, significantly and positively correlated to ROD21_RE_ at 1 dpi, and significantly and negatively correlated to the ROD21_RE_ at 7 dpi (Figure 3A_2_,A_3_). From the ileum, the association analysis showed no significant correlations of the H/L ratio with the ROD21 and the ROD21_RE_ during the infective cycle (Figure 3B_2_,B_3_). In the liver, the correlations analysis revealed that the H/L ratio was significantly and positively correlated to the ROD21 at 7 dpi and significantly and positively correlated to the ROD21_RE_ at the same day post-infection (Figure 3C_2_,C_3_).

### 2.4. Effect of Salmonella enteritidis Infection on IL-1β, IL-8, and IFN-γ Blood Serum Concentration, and Association with H/L Ratio

**Figure 4:**
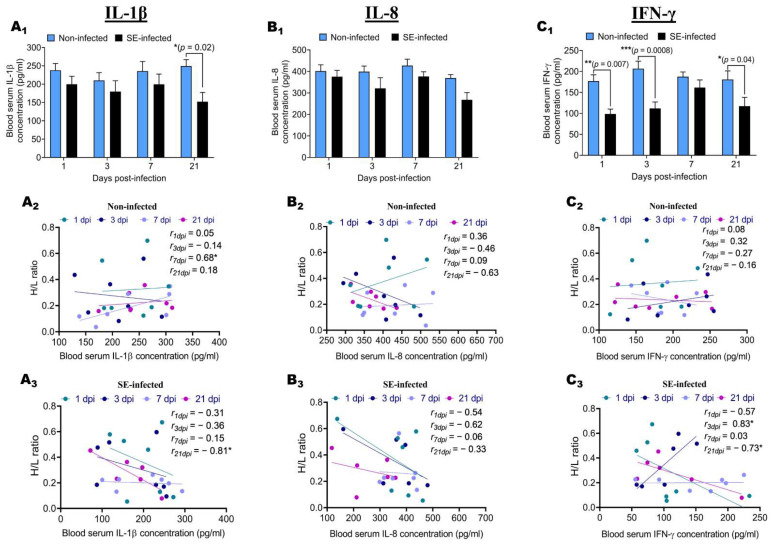
Determination of blood serum IL-1β, IL-8, and IFN-γ concentration during the infective cycle and association with the H/L ratio. After infection, the quantification of IL-1β (**A_1_**), IL-8 (**B_1_**), and IFN-γ (**C_1_**) in the blood serum was performed using enzyme-linked immunosorbent assays (ELISAs) (six to seven birds were used per group at each time point). (**A_2_**,**A_3_**) Correlation between H/L ratio and IL-1 β blood serum concentration in non-infected and SE-infected, respectively. (**B_2_**,**B_3_**) Correlation between H/L ratio and IL-8 blood serum concentration in non-infected and SE-infected, respectively. (**C_2_**,**C_3_**) Correlation between H/L ratio and IFN-γ blood serum concentration in non-infected and SE-infected, respectively. One-way ANOVA with Tukey’s post-test (α = 0.05) was used to compare different time points post-infection, and the results showed no significant differences for the three cytokines. Comparisons between the two groups during the infective cycle were performed by two-way ANOVA with Sidak’s post-test α = 0.05; correlations between the H/L ratio and the blood serum immune indicator were analyzed by Pearson correlation with Student’s *t*-test (one tail). * *p* < 0.05, ** *p* < 0.01 and *** *p* < 0.001.

To assess the effects of the *Salmonella enteritidis* challenge on the inflammatory response and to determine the relationship between the level of the inflammatory response and the H/L ratio, IL-1β, IL-8, and IFN-γ blood serum concentrations of NI and SE groups were measured by ELISA at 1, 3, 7, and 21 dpi (Figure 4). The results showed that the SE challenge induced a decrease in the IL-1β, IL-8, and IFN-γ blood serum concentration during the infective cycle (Figure 4A_1_,B_1_,C_1_). At 1, 3, and 7 dpi, no significant differences were found between NI and SE chickens in the concentration of IL-1β and IL-8 (Figure 4A_1_,B_1_), except for the concentration of IL-1β at 21 dpi, which was significantly (*p* = 0.02) increased in NI chickens compared to SE chickens (Figure 4A_1_). Similarly, at 7 dpi, no significant difference was found between NI and SE chickens in the concentration of IFN-γ (Figure 4C_1_). However, at 1, 3, and 21 dpi, the concentrations of IFN-γ were significantly (*p* = 0.007, *p* = 0.0008, and *p* = 0.04, respectively) increased in NI chickens compared to SE chickens. It was noteworthy that the IFN-γ blood serum concentration from NI chickens was picked at 3 dpi, whereas SE chickens were picked at 7 dpi. To verify the relationship between the H/L ratio and the intensity of the inflammatory response, we performed Pearson’s correlations between the H/L ratio and the IL-1β, IL-8, and IFN-γ blood serum concentration for each group at 1, 3, 7, and 21 dpi. In NI chickens, no significant correlations were observed between the H/L ratio and the IL-1β, IL-8, and IFN-γ blood serum concentration during the infective cycle, except at 7 dpi, where the concentration in IL-1β was significantly and positively correlated (r = 0.68; *p* < 0.05) to the H/L ratio (Figure 4A_2_,B_2_,C_2_). In comparison to the NI chickens, the SE chickens IL-1β, IL-8, and IFN-γ blood serum concentrations were negatively correlated to the H/L ratio, except for at 3 dpi, where the concentration of IFN-γ was significantly and positively correlated (r = 0.83; *p* < 0.05) to the H/L ratio (Figure 4A_3_,B_3_,C_3_). We observed that, at 21 dpi, the concentration in IL-1β and IFN-γ was significantly and negatively correlated (r = −0.81 and *p* < 0.05, r = −0.73; *p* < 0.05, respectively) to the H/L ratio (Figure 4A_3_,C_3_).

## 3. Discussion

*Salmonella* remains a significant threat to the poultry industry and public health. Food safety issues in poultry production associated with *Salmonella* contamination or infection represent substantial challenges. In addition, the health status of the live broiler flocks entering the processing facility can affect poultry food safety [1]. There is thus a critical urgency to find practical alternatives for the limitation of the risk associated with food-borne pathogens such as *Salmonella*. Emerging evidence suggested that the H/L ratio helped to select stress-, disease-, and *Salmonella typhimurium*-resistant chickens [18,19]. Moreover, the H/L ratio has been reported as highly heritable in chickens and wild avian species [19,20,21,25], indicating that this trait should respond efficiently to the selection [26]. However, despite this wide range of link shreds of evidence between the H/L ratio and the disease resistance, information related to the H/L ratio and the resistance to *Salmonella enteritidis* infection in the chicken is scarce. Therefore, this study performed the first association analysis of the H/L ratio with the resistance and inflammatory response to *Salmonella enteritidis*. *Salmonella* infections in broiler chickens have been linked to a decreased production performance, intestinal colonization, inflammatory reactions, and internal organ invasion [27]. In the present study, we found that the *Salmonella enteritidis* challenge decreased the live weight during the infective cycle. These results are consistent with the previous studies [28,29]. In contrast with the present study, Sharkawy and collaborators [30] reported high mortalities, inappetence, and the manifestation of respiratory problems under *Salmonella typhimurium* infection in chicken, indicating that the strains used in the current work could be the cause of the reduced mortality obtained.

*Salmonella enteritidis* infection in chicken is a serious concern to the poultry business, since it looks to be a source of contamination not only for other chickens in co-housing rearing facilities but also for the global human population via the consumption of contaminated products [31]. Three possible illness outcomes are possible, depending on the host’s resistance and immune response competency, as well as the infecting serovars: acute/fatal salmonellosis, chronic salmonellosis, or bacterial clearance [32,33]. *Salmonella enteritidis* is one of the most common causes of food-borne disease [34,35]. In the current study, we assessed the colonization and persistence of *Salmonella enteritidis* in the blood, liver, ileum, and caecum of orally infected chicken during the infective cycle. Our results showed that the bacterial load in the blood and different tissues differed significantly across the infective cycle. It was noteworthy that the bacterial load was significantly increased in the intestine compared to that in the blood and liver, which remained approximatively constant during the infective cycle. We observed that the bacterial load in the caecum decreased with time, whereas, in the ileum, it increased. These results suggest that *Salmonella enteritidis* colonization increases in the small intestine and causes successful deep organ colonization and systemic infection. Our findings follow the results reported by Pardo-Roa and colleagues [14]. By contrast, Mon and associates demonstrated that *Salmonella* bacterial colonization was restricted to the caecum, with no bacterial burden detected in the systemic organs of the infected chicks’ spleen or liver. This discrepancy can be attributable to the technique utilized in our investigation for detecting and quantifying *Salmonella*. A recent study validates the sensitivity and accuracy of Q-PCR quantification, demonstrating that it is an appropriate test for quantifying a viable and non-viable *Salmonella* load in a chicken setting [36]. An association analysis of the H/L ratio with the bacterial load in the blood and different tissues revealed no significant correlations between the H/L ratio and *Salmonella enteritidis* load in the blood and liver tissue during the infective cycle. However, significant correlations were observed between the H/L ratio and the bacterial load in different gut segments at 7 dpi. The H/L ratio measured at 7 days old was significantly and negatively correlated to the bacterial load recorded at 7 dpi in the ileum, whereas it was significantly and positively correlated to that of the caecum at the same time point post-infection. Together, these results suggest the existence of a relationship between the H/L ratio and the colonization resistance. Specifically, these results indicate differential *Salmonella enteritidis* colonization resistances between chickens with low and high H/L ratios and specific- H/L- tissue resistance.

Pathogenicity islands are vast clusters of genes found in the genomes of pathogenic bacteria but not in the genomes of non-pathogenic strains of the same or closely related species [37]. Numerous PAIs can excise from the chromosome and re-integrate into it [38]. Region of difference 21 (ROD21) is one of the excisable *Salmonella enterica* PAIs [13]. It was initially discovered in the chromosomes of *Salmonella enteritidis*, *Salmonella gallinarum*, *Salmonella dublin*, and *Salmonella typhi* [12,39], but was recently discovered in other serovars, such as *Salmonella pullorum*, *Salmonella nitra*, and *Salmonella antatum* [40]. *Salmonella enteritidis* strains deficient in ROD21 cannot invade the liver and spleen of mice or chickens [13,41]. Notably, Pardo-Roa and colleagues hypothesized that ROD21 excision is a genetic mechanism necessary for *Salmonella enteritidis* to successfully invade the intestinal epithelium and cause systemic infection in mice [14]. We proposed that a high excision of ROD21 is associated with susceptibility to *Salmonella enteritidis* and a weak intestinal immune barrier in chicken. We described here, for the first time, the dynamic of ROD21 excision in the gastrointestinal tract (Caecum and Ileum) and liver of *Salmonella enteritidis*-challenged chicken during the infective cycle. We found that the frequency of ROD21 excision was significantly increased in the caecum and ileum at the early stages of the infective cycle. In contrast, the frequency of ROD21 excision remained constant and low in the liver compared to that of the intestine. These findings corroborate the findings of Pardo-Roa and colleagues. They found that ROD21 excision significantly increases the bacterial population colonizing mesenteric lymph nodes during the early stages of the infectious cycle (before 48 h post-infection) while remaining extremely low in the liver and spleen during these stages [14]. The same authors found that the relative ROD21 excision varied from 0.3 to 0.7, whereas, in our study, we reported a variation from 0.75 to 1.04. This result suggests that the excision of ROD21 PAIs occurs at a high frequency in chicken compared to mice. In the caecum, the associative analysis revealed that the H/L ratio was significantly and positively correlated to the ROD21 relative excision at the early stage of the infection (1 dpi). In comparison, it was significantly and negatively correlated at the later stage post-infection (7 dpi). The change in the correlation from positive to negative with time suggests that chickens with a high H/L ratio were more susceptible at the earlier stage post-infection, whereas, at the later stage, chickens with a low H/L ratio were (i) more susceptible or (ii) more resistant and displayed an enhanced immune response or environmental conditions that induced *Salmonella* to increase the ROD21 excision. Salazar-Echegarai and colleagues suggested that, during the infective cycle, the exposure of *Salmonella* to reactive oxygen species or pH changes can modulate the excision of ROD21 [42]. This correlation sign change with the time of and difference in ROD21 excision between individuals with a high and low H/L ratio can be attributed to a possible differential cecal microbiota composition and functional capacity.

In the liver, the absolute and relative excision of ROD21 were significantly and positively correlated to the H/L ratio at 7 dpi, suggesting that, at this stage of the infective cycle, chickens with a high H/L ratio display a high frequency of ROD21 excision, have a weak intestinal epithelium barrier, and were more susceptible. Our experimental results demonstrate that chickens with a low H/L ratio (<0.3) are more resistant to the chickens with a high H/L ratio (>0.3). However, environmental factors impact the ROD21 excision frequency [14] and increase when *Salmonella enteritidis* affects dendritic cells and macrophages [13]. In addition, lysosome-like circumstances and a lower pH boost the ROD21 PAI frequency and transmission [42]. Pardo-Roa and colleagues found a high level of ROD21 excision in the mesenteric lymph node at the early stage of the infective cycle in mice and suggested that the environmental conditions found in this site at the initial stages of infection promote ROD21 excision [14]. Knowing that the phagosome environment, such as a low pH, high temperatures, and high amounts of reactive oxygen species, induce an increase in ROD21 excision, the same authors thought it was possible to propose that only bacteria that have undergone ROD21 excision can survive intracellularly in the ileum and colon and can reach/colonize, in an excised state, the mesenteric lymph node [14].

Inflammatory and immunological responses rely on cytokines, which cells produce [22]. These chemokines influence immune cells’ proliferation, differentiation, and activity [43]. Both interferon-gamma and interleukin play a critical role in promoting Th1 immune responses, which are essential for promoting protective responses against invading pathogens [44]. To better understand the relationship between the H/L ratio and the level of the inflammatory response in the blood, we investigated the effects of the *Salmonella* challenge on the levels of the IL-1β, IL-8, and IFN-γ blood serum concentration of NI and SE groups at 1, 3, 7, and 21 dpi. Then, we established the link between the H/L ratio and the IL-1β, IL-8, and IFN-γ blood serum concentration under normal and infected conditions during the infective cycle. In the present work, the *Salmonella enteritidis* challenge decreased the IL-1β, IL-8, and IFN-γ blood serum concentration during the infective cycle.

Moreover, the correlations analysis revealed that the H/L ratio is associated with the level of IL-1β and IFN-γ blood serum concentration at 21 dpi. In general, the H/L ratio was negatively correlated with these cytokines and chemokines at specific stages of the infectious cycle, indicating that chickens with a low H/L ratio have a more robust and appropriate inflammatory response to *Salmonella enteritidis* infection than chickens with a high H/L ratio. Certain variables, such as the lack of premature gut flora and an undeveloped immune system, lead to early infection. In comparison, older birds are less vulnerable to infection because their immune systems and gut flora are well established and compete for receptors or create metabolic products (bacteriocins) that inhibit *Salmonella* development [45,46]. An increased heterophil influx to the infection site contributes to an increased resistance against systemic *Salmonella enteritidis* infections in neonatal chickens [47]. In addition, an upregulation of proinflammatory cytokines IL-1b, IL-6, and IL-8 in an experiment including heterophils cells stimulated by *Salmonella enteritidis* has been reported by Kogut et al. [48]. According to our results and the pre-cited reports, it is possible to suggest that chickens with a low H/L ratio display a reduced heterophil cells number but with an enhanced function of this particular avian immune cell.

In conjunction with the previous, the current study supports the conclusion that the H/L ratio is a marker of robustness and a criterion for selecting for both general resistance and *Salmonella* resistance in chicken. Therefore, it could be concluded that *Salmonella enteritidis* affects the chickens’ performances and immune responses, but the chickens with a low H/L ratio are less affected by the infection and display increased inflammatory responses under normal and infected conditions. Significant links were observed between the H/L ratio measured at 7 days old and some essential traits, such as the colonization resistance and inflammatory response, during the chicks’ developmental cycle under both pre-cited conditions. The H/L ratio and the colonization resistance to *Salmonella enteritidis* were mainly correlated at 7 dpi. A correlation between the H/L ratio and the inflammatory response was found at 21 dpi under *Salmonella* infection. It will be interesting to elucidate the molecular and genetic mechanisms behind those significant associations by utilizing modern equipment in the field of omics and next-generation sequencing research.

## 4. Materials and Methods

### 4.1. Animals and Experimental Design

In this study, 300 Jinxing yellow chickens H/L line freshly hatched (IAS-CAAS, Beijing, China) were involved in a 21-day *Salmonella enteritidis* challenge experiment. At 1 day old, the chicks were immediately transferred to housing rooms equipped with sterilized isolation ventilated cages (IVC) (IPQ-type 3 negative pressure isolator). The birds were randomly divided into two groups: non-infected (NI) and SE-infected (SE). After *Salmonella* infection, the birds of each group were assigned to one IVC with an average of 100 and 200 birds, respectively, for NI and SE groups. Two days before the assignment of birds to each treatment, the temperature in the IVC was maintained at 37 °C. Then, it was fixed at 35 °C with a weekly decrease of 2 °C until the experiment ended (21 days post-infection). The chicks received ad libitum Specific Pathogen Free (SPF) feed (Beijing Keao Xieli Feed Co., Ltd., Beijing, China) and open access to sterilized water throughout the experiment.

### 4.2. Determination of the H/L Ratio

At 7 days old, 10 µL of fresh blood samples was collected from the basilic vein of each bird. The blood was taken and smeared on microscopic glass slides using a syringe, a needle, and a micropipette of 10 µL to drop the same blood volume. The resulting blood smears were air-dried and then stained using Giemsa staining. One hundred leukocytes were counted, including heterophils, lymphocytes, and monocytes, following a schematic diagram and using a Leica DM500 microscope with a magnification of 100× [49]. The H/L ratio was calculated by dividing the number of heterophil cells by lymphocyte cells.

### 4.3. Salmonella Challenge and Sampling

*Salmonella enteritidis* 50335 (Institute of Veterinary drugs Control, Beijing, China) was used to challenge the birds in this experiment. After resuscitation and growth of the bacteria at 37 °C in Luria Bertani Broth (LB) with agitation (150 rpm) overnight, concentrates were resuspended in sterile phosphate-buffered saline (PBS). The final number of colony-forming units (CFUs) was determined by plating in triplicate ten times serial dilutions on Brilliant Green agar (37 °C, overnight). Before infection, all of the chicks were checked for *Salmonella* presence by culturing cloacal swab samples in buffered peptone water overnight at 37 °C with agitation. According to the results, no infected chicks were detected. At 7 days old, the birds from the SE group were infected by oral gavage with 1 mL of PBS containing 1 × 10^10^ CFUs of *Salmonella enteritidis*. The birds from the NI group received the same volume of sterile PBS. At 1, 3, 7, and 21 days post-infection (dpi), 20 chickens from each experimental group were randomly selected and slaughtered. At each dpi before slaughter, the chicks were weighed, and blood samples were collected in double: one tube for the collection of serum, and the other stored at −20 °C for DNA extraction.

### 4.4. DNA Extraction

The genomic DNA (gDNA) used in the current study was purified using the Phenol-Chloroform method. Briefly, blood (20 µL) and cells suspended from homogenized organs (30 mg) were mixed with 800 µL of Lysis buffer (Tris 6.43%, EDTA 4.94%, NaCl 62.07%, SDS 26.56%), 4 µL of RNase A (Tiangen, Beijing, China), and 25 µL of proteinase k. The mixture was shaken vigorously by inversion for 10 min, and then incubated at 56 °C overnight. Next, the DNA extraction procedure was carried out by adding an equal volume of phenol, chloroform, and absolute ethanol and gently shaking the solution until it became milky. After centrifuging the mixture at 12,000 rpm for ten minutes, the organic phase was removed. Next, 1000 µL of absolute ethanol was used to precipitate the gDNA. Finally, the DNA pellet was washed with 300 µL of 75% alcohol (ethanol). At the last step, the tubes were centrifuged at 8500 rpm for 5min; then, the supernatant was removed, and the pellet was air-dried for 5 min at room temperature. Finally, the pellet of DNA was resuspended in 200 µL of nuclease-free water (double distilled water).

### 4.5. Quantitative Real-time PCR (RT-QPCR)

**Figure 5:**
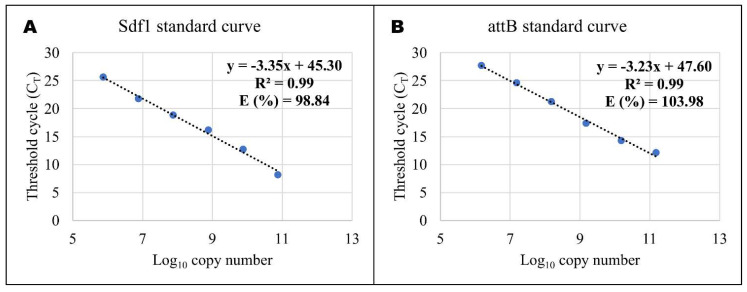
Detection limit and reproducibility of qPCR on *Salmonella enteritidis* Sdf1 and attB genes: (**A**) Sdf1 standard curve generated from quantification cycle numbers of a 10-fold dilution series of an amplicon product of 103 bp cloned into a pLB plasmid vector (Lethal Based Fast Cloning kit) plasmid vector in DNase- and RNase-free water (from 10^0^ to 10^6^). (**B**) Sdf1 standard curve generated from quantification cycle numbers of a 10-fold dilution series of an amplicon product of 199 bp cloned into a pLB plasmid vector (Lethal Based Fast Cloning kit) in DNase- and RNase-free water (from 10^0^ to 10^6^).

To determine the bacterial (*Salmonella enteritidis*) load and the *Salmonella enteritidis* region of difference 21 (ROD21) excision throughout the infective cycle in the blood, liver, ileum, and caecum, gDNA of 10–12 birds from each group were used. Quantitative real-time PCR (Q-PCR) was performed using TaqMan probes and KAPA PROBE FAST qPCR Master Mix (2×) Kit (KAPA BIOSYSTEMS, US Wilmington, Mass, USA: Wilmington, NC, USA), according to the manufacturer’s instructions, for volume total of 20 µL of the reaction mixture. Primers and fluorescent probes (5′FAM-TAMRA3′) for *Salmonella* differentiating fragment 1 (Sdf1) and attB [14] were used in this study (Table 1). To quantify the copy number in different organs, we used a standard curve for Sdf1 (Figure 5A) and attB (Figure 5B). The amplification system was as follows: 95 °C for 30 s, 40 cycles of 95 °C 5 s, and 60 °C for 34 s, with an additional step of 60 °C for 15 s at the end.

**Table 1:** Primers and probe of Sdf1 and attB sequences.

Genes	Forward (F) Reverse (R) Primers and Probe (P) 5′ to 3′	Accession no./Reference
Sdf1	F: TCCCTGAATCTGAGAAAGAAAAACTC	No.AF370707.1
	R: TTGATGTGGTTGGTTCGTCACT	
	P: TGCAGCGAGCATGTTCTGGAAAGC	
attB	F: GTTACTATGCGCCCCGTTCACAC	[14,50]
	R: CCGATTAAGCCCCAAAAACTATG	
	P: TTCGAGTCCAGTCAGAGGA	

### 4.6. Blood Serum IL-1β, IL-8, and IFN-γ Concentrations

Eight chickens from each of the two groups were used at each time point post-infection (1, 3, 7, and 21 dpi). The IL-1β, IL-8, and IFN-γ concentrations in the serum were measured using enzyme-linked immunosorbent assay (ELISA) kits, according to the manufacturer’s instructions (Cusabio Biotech Co., Ltd., Wuhan, China). In brief, serum was diluted (100-fold) for generation of a standard curve with horseradish peroxidase (HRP) and conjugated antibody for the targeted immune factor was added to a plate precoated with the target. After incubation and wash, the intensity of the color generated due to the addition of a substrate solution was measured by a microplate reader. Based on the standard curves, the concentration of IL-1β, IL-8, and IFN-γ in the serum was calculated.

### 4.7. Statistical Analysis

GraphPad Prism version 8 (GraphPad Software, San Diego, CA, USA) and R version 4.0.5 (2021-03-31) were used to analyze the data. The changes in body weight, bacterial load (Sdf1), ROD21 absolute (ROD21), relative (ROD21_RE_), and the blood serum cytokines and chemokines concentrations in serum during the infective cycle were analyzed using two-way ANOVA with Sidak’s multiple comparisons in GraphPad Prism. The differences in bacterial load among the blood, liver, ileum, and caecum at each time were analyzed using one-way ANOVA with Tukey’s post-test in R. Correlations between 2 factors were performed using Pearson’s correlation in GraphPad Prism, with the significance determined by the Student *t*-test. Statistical significance was declared when the *p*-value was <0.05. The results are expressed as the mean and standard error of the mean (SEM).

## Data Availability

The datasets generated during and/or analyzed during the current study are available from the corresponding author on reasonable request.

## References

[1] Ricke S.C. (2021). Strategies to Improve Poultry Food Safety, a Landscape Review. Annu. Rev. Anim. Biosci..

[2] Foley S.L., Johnson T.J., Ricke S.C., Nayak R., Danzeisen J. (2013). Salmonella pathogenicity and host adaptation in chicken-associated serovars. Microbiol. Mol. Biol. Rev..

[3] Park S.Y., Woodward C.L., Kubena L.F., Nisbet D.J., Birkhold S.G., Ricke S.C. (2008). Environmental Dissemination of Foodborne Salmonella in Preharvest Poultry Production: Reservoirs, Critical Factors, and Research Strategies. Crit. Rev. Environ. Sci. Technol..

[4] Castillo N.A., de Moreno de LeBlanc A., Galdeano C.M., Perdigón G. (2012). Probiotics: An alternative strategy for combating salmonellosis: Immune mechanisms involved. Food Res. Int..

[5] Peruzy M.F., Capuano F., Proroga Y.T.R., Cristiano D., Carullo M.R., Murru N. (2020). Antimicrobial Susceptibility Testing for Salmonella Serovars Isolated from Food Samples: Five-Year Monitoring (2015–2019). Antibiotics.

[6] Van Immerseel F., Cauwerts K., Devriese L.A., Haesebrouck F., Ducatelle R. (2002). Feed additives to control Salmonella in poultry. World’s Poult. Sci. J..

[7] Applegate T.J., Klose V., Steiner T., Ganner A., Schatzmayr G. (2010). Probiotics and phytogenics for poultry: Myth or reality?. J. Appl. Poult. Res..

[8] Hume M.E. (2011). Historic perspective: Prebiotics, probiotics, and other alternatives to antibiotics. Poult. Sci..

[9] Ricke S.C. (2018). Impact of Prebiotics on Poultry Production and Food Safety. Yale J. Biol. Med..

[10] Ricke S.C., Lee S.I., Kim S.A., Park S.H., Shi Z. (2020). Prebiotics and the poultry gastrointestinal tract microbiome. Poult. Sci..

[11] Festa R., Ambrosio R.L., Lamas A., Gratino L., Palmieri G., Franco C.M., Cepeda A., Anastasio A. (2021). A Study on the Antimicrobial and Antibiofilm Peptide 1018-K6 as Potential Alternative to Antibiotics against Food-Pathogen Salmonella enterica. Foods.

[12] Thomson N.R., Clayton D.J., Windhorst D., Vernikos G., Davidson S., Churcher C., Quail M.A., Stevens M., Jones M.A., Watson M. (2008). Comparative genome analysis of Salmonella Enteritidis PT4 and Salmonella Gallinarum 287/91 provides insights into evolutionary and host adaptation pathways. Genome Res..

[13] Quiroz T.S., Nieto P.A., Tobar H.E., Salazar-Echegarai F.J., Lizana R.J., Quezada C.P., Santiviago C.A., Araya D.V., Riedel C.A., Kalergis A.M. (2011). Excision of an unstable pathogenicity island in Salmonella enterica serovar Enteritidis is induced during infection of phagocytic cells. PLoS ONE.

[14] Pardo-Roa C., Salazar G.A., Noguera L.P., Salazar-Echegarai F.J., Vallejos O.P., Suazo I.D., Schultz B.M., Coronado-Arrazola I., Kalergis A.M., Bueno S.M. (2019). Pathogenicity island excision during an infection by Salmonella enterica serovar Enteritidis is required for crossing the intestinal epithelial barrier in mice to cause systemic infection. PLoS Pathog..

[15] Minias P. (2019). Evolution of heterophil/lymphocyte ratios in response to ecological and life-history traits: A comparative analysis across the avian tree of life. J. Anim. Ecol..

[16] Lentfer T.L., Pendl H., Gebhardt-Henrich S.G., Frohlich E.K., Von Borell E. (2015). H/L ratio as a measurement of stress in laying hens—Methodology and reliability. Br. Poult. Sci..

[17] Thiam M., Barreto Sánchez A.L., Zhang J., Zheng M., Wen J., Zhao G., Wang Q. (2021). Association of Heterophil/Lymphocyte Ratio with Intestinal Barrier Function and Immune Response to Salmonella enteritidis Infection in Chicken. Animals.

[18] al-Murrani W.K., Kassab A., al-Sam H.Z., al-Athari A.M. (1997). Heterophil/lymphocyte ratio as a selection criterion for heat resistance in domestic fowls. Br. Poult. Sci..

[19] Al-Murrani W.K., Al-Rawi A.J., Al-Hadithi M.F., Al-Tikriti B. (2006). Association between heterophil/lymphocyte ratio, a marker of ‘resistance’ to stress, and some production and fitness traits in chickens. Br. Poult. Sci..

[20] Campo J.L., Davila S.G. (2002). Estimation of heritability for heterophil:lymphocyte ratio in chickens by restricted maximum likelihood. Effects of age, sex, and crossing. Poult. Sci..

[21] Wilcoxen T.E., Boughton R.K., Morgan G.M., Schoech S.J. (2013). Heritability of immunological characteristics in Florida Scrub-Jays (Aphelocoma coerulescens). Can. J. Zool..

[22] Dar M.A., Urwat U., Ahmad S.M., Ahmad R., Kashoo Z.A., Dar T.A., Bhat S.A., Mumtaz P.T., Shabir N., Shah R.A. (2019). Gene expression and antibody response in chicken against Salmonella Typhimurium challenge. Poult. Sci..

[23] Quinteiro-Filho W.M., Calefi A.S., Cruz D.S.G., Aloia T.P.A., Zager A., Astolfi-Ferreira C.S., Piantino Ferreira J.A., Sharif S., Palermo-Neto J. (2017). Heat stress decreases expression of the cytokines, avian beta-defensins 4 and 6 and Toll-like receptor 2 in broiler chickens infected with Salmonella Enteritidis. Vet. Immunol. Immunopathol..

[24] Song J., Li Q., Everaert N., Liu R., Zheng M., Zhao G., Wen J. (2020). Dietary Inulin Supplementation Modulates Short-Chain Fatty Acid Levels and Cecum Microbiota Composition and Function in Chickens Infected With Salmonella. Front. Microbiol..

[25] Zhang J., Wang J., Li Q., Wang Q., Wen J., Zhao G. (2020). Comparison of the Efficiency of BLUP and GBLUP in Genomic Prediction of Immune Traits in Chickens. Animals.

[26] Zhu B., Li Q., Liu R., Zheng M., Wen J., Zhao G. (2019). Genome-Wide Association Study of H/L Traits in Chicken. Animals.

[27] Chalghoumi R., Marcq C., Thewis A., Portetelle D., Beckers Y. (2009). Effects of feed supplementation with specific hen egg yolk antibody (immunoglobin Y) on Salmonella species cecal colonization and growth performances of challenged broiler chickens. Poult. Sci..

[28] Al-Murrani W.K., Al-Rawi I.K., Raof N.M. (2002). Genetic resistance to Salmonella typhimurium in two lines of chickens selected as resistant and sensitive on the basis of heterophil/lymphocyte ratio. Br. Poult. Sci..

[29] Wu Q.J., Zheng X.C., Wang T., Zhang T.Y. (2018). Effect of dietary oridonin supplementation on growth performance, gut health, and immune response of broilers infected with Salmonella pullorum. Ir. Vet. J..

[30] El-Sharkawy H., Tahoun A., El-Gohary A.E.A., El-Abasy M., El-Khayat F., Gillespie T., Kitade Y., Hafez H.M., Neubauer H., El-Adawy H. (2017). Epidemiological, molecular characterization and antibiotic resistance of Salmonella enterica serovars isolated from chicken farms in Egypt. Gut Pathog..

[31] Mon K.K.Z., Zhu Y., Chanthavixay G., Kern C., Zhou H. (2020). Integrative analysis of gut microbiome and metabolites revealed novel mechanisms of intestinal Salmonella carriage in chicken. Sci. Rep..

[32] Chappell L., Kaiser P., Barrow P., Jones M.A., Johnston C., Wigley P. (2009). The immunobiology of avian systemic salmonellosis. Vet. Immunol. Immunopathol..

[33] Wales A.D., Davies R.H. (2011). A critical review of Salmonella Typhimurium infection in laying hens. Avian Pathol..

[34] Cummings P.L., Kuo T., Javanbakht M., Shafir S., Wang M., Sorvillo F. (2016). Salmonellosis Hospitalizations in the United States: Associated Chronic Conditions, Costs, and Hospital Outcomes, 2011, Trends 2000–2011. Foodborne Pathog. Dis..

[35] Dewey-Mattia D., Manikonda K., Hall A.J., Wise M.E., Crowe S.J. (2018). Surveillance for Foodborne Disease Outbreaks—United States, 2009-2015. MMWR Surveill. Summ..

[36] Zhang J., Khan S., Chousalkar K.K. (2020). Development of PMAxxTM-Based qPCR for the Quantification of Viable and Non-viable Load of Salmonella From Poultry Environment. Front. Microbiol..

[37] Gal-Mor O., Finlay B.B. (2006). Pathogenicity islands: A molecular toolbox for bacterial virulence. Cell Microbiol..

[38] Nieto P.A., Pardo-Roa C., Salazar-Echegarai F.J., Tobar H.E., Coronado-Arrazola I., Riedel C.A., Kalergis A.M., Bueno S.M. (2016). New insights about excisable pathogenicity islands in Salmonella and their contribution to virulence. Microbes Infect..

[39] Porwollik S., Santiviago C.A., Cheng P., Florea L., Jackson S., McClelland M. (2005). Differences in gene content between Salmonella enterica serovar Enteritidis isolates and comparison to closely related serovars Gallinarum and Dublin. J. Bacteriol..

[40] Piña-Iturbe A., Ulloa-Allendes D., Pardo-Roa C., Coronado-Arrázola I., Salazar-Echegarai F.J., Sclavi B., González P.A., Bueno S.M. (2018). Comparative and phylogenetic analysis of a novel family of Enterobacteriaceae-associated genomic islands that share a conserved excision/integration module. Sci. Rep..

[41] Feasey N.A., Hadfield J., Keddy K.H., Dallman T.J., Jacobs J., Deng X., Wigley P., Barquist L., Langridge G.C., Feltwell T. (2016). Distinct Salmonella Enteritidis lineages associated with enterocolitis in high-income settings and invasive disease in low-income settings. Nat. Genet..

[42] Salazar-Echegarai F.J., Tobar H.E., Nieto P.A., Riedel C.A., Bueno S.M. (2014). Conjugal transfer of the pathogenicity island ROD21 in Salmonella enterica serovar Enteritidis depends on environmental conditions. PLoS ONE.

[43] Kaiser P., Poh T.Y., Rothwell L., Avery S., Balu S., Pathania U.S., Hughes S., Goodchild M., Morrell S., Watson M. (2005). A genomic analysis of chicken cytokines and chemokines. J. Interferon Cytokine Res..

[44] Eckmann L., Kagnoff M.F. (2001). Cytokines in host defense against Salmonella. Microbes Infect..

[45] Beal R.K., Wigley P., Powers C., Hulme S.D., Barrow P.A., Smith A.L. (2004). Age at primary infection with Salmonella enterica serovar Typhimurium in the chicken influences persistence of infection and subsequent immunity to re-challenge. Vet. Immunol. Immunopathol..

[46] Crhanova M., Hradecka H., Faldynova M., Matulova M., Havlickova H., Sisak F., Rychlik I. (2011). Immune response of chicken gut to natural colonization by gut microflora and to Salmonella enterica serovar enteritidis infection. Infect. Immun..

[47] Swaggerty C.L., Kogut M.H., Ferro P.J., Rothwell L., Pevzner I.Y., Kaiser P. (2004). Differential cytokine mRNA expression in heterophils isolated from Salmonella-resistant and -susceptible chickens. Immunology.

[48] Kogut M.H., Rothwell L., Kaiser P. (2003). Differential regulation of cytokine gene expression by avian heterophils during receptor-mediated phagocytosis of opsonized and nonopsonized Salmonella enteritidis. J. Interferon Cytokine Res..

[49] Fidan E.D., Nazligül A., Türkyilmaz M., Aypak S.U., Kilimci F.S., Karaarslan S., Kaya M.J.R.B.D.Z. (2017). Effect of photoperiod length and light intensity on some welfare criteria, carcass, and meat quality characteristics in broilers. Rev. Bras. De Zootec..

[50] Bueno S.M., Santiviago C.A., Murillo A.A., Fuentes J.A., Trombert A.N., Rodas P.I., Youderian P., Mora G.C. (2004). Precise excision of the large pathogenicity island, SPI7, in Salmonella enterica serovar Typhi. J. Bacteriol..

